# On Tests for Arsenic, in Answer to Mr. Hume's Paper in Our Last Number

**Published:** 1818-03

**Authors:** James Kerr


*For the London Medical and Physical Journal.*

### *On Tests for Arsenic, in Answer to Mr. Hume's Paper in our last number;* by JAMES KERR, Esq.

I AM very glad to find, that my paper, in your Journal for August last, has, at length, *drawn out* Mr. Hume in this month's, on the subject of tests for arsenic. My object will now be attained—to bring the subject before your readers, and to have its discussion conducted by one so well qualified as Mr. Hume is, and whose claim to the discovery of the silver test is so well established. I wish I could think as highly of Mr. Hume's politeness as of his science; but he has been so *liberal* of his censures, and so *illiberal* in his insinuations, that a superficial observer might suppose, that Dr. Neale, Aminicus, and myself, had entered into a conspiracy, that his Majesty's liege subjects might be poisoned with impunity. I will not pay those gentlemen so bad a compliment as to enter on their defence, but will venture to say, that, whatever errors we labour under, the motive of us all has been, a desire to be satisfied in our minds of the relative value of each test, and what is its real weight in the scale of corroborative testimony.

Mr. Hume thus begins his paper, "More than twenty-eight years have now elapsed since I first discovered the excellence of silver as a test for arsenic. This, like all other discoveries, has been, especially under my own guidance, gradually improving, through the aid of experiments founded on observation, and called for by numberless objections of the most absurd or frivolous nature made by others." There is no doubt, from the general tenor of Mr. Hume's paper, that I am included in the list of absurd or frivolous objectors: but one consolation remains,—that, from Mr. Hume's own acknowledgment, I may lay claim to some merit in the improvements that may hereafter result from his renewed labours, and may say with Falstaff,—"I tell thee what, Hal, I'm not only witty in myself, but the *cause* of wit in others."—I must apologise to your readers for troubling them with my own personal feelings, and proceed to the subject. Mr. Hume says, "I beg to ask, why one of the tests which I have recommended—the ammoniaco-nitrate of silver, was not taken into Mr. Kerr's or Dr. Neale's consideration, on following up, as is pretended, the experiments of Dr. Edwards?" To this I answer, that I did not then know of the improvement on the original discovery, and the time of a medical man is too much broken in upon to admit of long research; besides, I professed merely to repeat the two experiments of Dr. Neale, from the newspaper report. I have since followed Mr. Hume's advice, and carefully read all the papers he refers to, which I hope will convince him, that, although I was ignorant, I am not willing to remain so. I find, on using the ammoniaco-nitrate of silver, instead of the simple nitrate, that it produces no precipitate with the phosphate of soda; and that it turns a decoction of onions of a pale yellow, which gradually darkens to a fawn reddish brown, and in a few hours nearly black; whereas, Mr. Hume's statement leads us to think, it becomes "quite red," and remains so. On mixing some decoction of onions with a solution of phosphate, and dropping in the ammoniaco-nitrate, the fluid becomes slightly yellow and remains transparent. I also find that the simple nitrate of silver detects a much smaller portion of arsenic than it does of phosphate of soda; for, with 1 to 200,000 of the former, a very evident yellow precipitate takes places; but, with the latter, in 1 to 32,000, there was only a slight yellowish opacity. But, there is one question I would ask Mr. Hume,—When he improved the silver test by putting it into the form of ammoniaco-nitrate, was this done because he knew that the simple nitrate would form a yellow precipitate with phosphate of soda, or because it made the test a more delicate one? I much question, if Mr. H. knew, before the present agitation of the subject, that the ammoniaco-nitrate would form a yellow precipitate with the phosphate of soda.

The experiment of Dr. Ayrton Paris, quoted by Mr. Hume, I have repeated and found perfectly correct; but am surprised to see in Mr. Crowfoot's paper, in your Journal for last December, the following statement:—"Mr. K. considers the nitrate of silver as no longer admissible as a test for the arsenious acid, from the circumstance of the phosphate, as well as the arsenite of silver, being precipitated of a yellow colour. Upon the first precipitation of these two salts, I allow there is a great resemblance; but, if we suffer them to remain undisturbed and exposed to the light for a few hours, the difference will be observable—the former becoming gradually of a brown colour, while the latter assumes a blackish hue." But I cannot help thinking, that this is only a *lapsus*, and that the words *former* and *latter* are not placed as Mr. C. intended.—Besides, for my greatest fault with Mr. Hume (ignorance of the ammoniaco-nitrate), this reply of Mr. Crowfoot's furnishes me with this palliation,—that Mr. C. appears to be a fellow criminal, and, under the protection of his acknowledged abilities and information, I am glad to shelter myself. On repeating Mr. Crowfoot's experiment, I find that sulphuretted hydrogen causes no change in the phosphate of soda, but produces a very perceptible yellowness in a very weak solution of arsenious acid; thus retaining its character as a test, as far as phosphate of soda is concerned.

Before I finish my letter, I must express my strongest wishes that Mr. Hume would soon favour us with his recent enquiries into this subject, as he says, in his last paper, "the following remarks, although hastily drawn up, will serve, however, as a preface to a more extensive enquiry."

I can assure Mr. Hume, I have no wish to rob him of his well-earned fame, but shall at all times be ready and thankful to be set right by him, on this or any other subject; and that, when the public good, or Mr. Hume's just claims, are concerned, they shall be paramount with me to any personal consideration.

*Great Yarmouth; Jan.* 28, 1818.

Certain, as we are, that Mr. Hume could have none but the best intentions, we are concerned to find that his language has produced any unpleasant feelings. We hope, however, that Mr. Kerr's suggestion will remind our correspondents, how necessary it is, in all matters of controversy, to be more than ordinarily guarded in our expressions.

