# Erratum

**Published:** 1818-04

**Authors:** 


Erratum.—Page 191, line 10, "would form a yellow precipitate, &c." should have been "would *not* form, &c."

